# Letrozole cotreatment improves the follicular output rate in high-body-mass-index women with polycystic ovary syndrome undergoing IVF treatment

**DOI:** 10.3389/fendo.2023.1072170

**Published:** 2023-03-03

**Authors:** Yali Liu, Jiaying Lin, Xi Shen, Qianqian Zhu, Yanping Kuang

**Affiliations:** Department of Assisted Reproduction, Shanghai Ninth People’s Hospital Affiliated to Shanghai Jiaotong University School of Medicine, Shanghai, China

**Keywords:** letrozole, polycystic ovarian syndrome, high body mass index, progestin-primed ovarian stimulation, follicular output rate

## Abstract

**Background:**

Women who have polycystic ovary syndrome (PCOS) with high body mass index (BMI) typically have an attenuated ovarian response and decreased follicular size, which are linked to unfavourable clinical outcomes following *in vitro* fertilization (IVF) therapy. The follicular output rate (FORT), a qualitative indicator of follicular response, seems to be positively linked to the clinical outcomes of IVF. Progestin-primed ovarian stimulation (PPOS) has become an alternative to gonadotropin-releasing hormone (GnRH) analogues to inhibit the premature luteinizing hormone (LH) surge. As letrozole (LE) shows promise in enhancing ovarian response, we compared PPOS with and without LE for PCOS in high BMI women with a focus on the FORT and associated clinical and pregnancy outcomes.

**Methods:**

For the recruited 1508 women, ten variables including AFC; age; basal sex hormone level; BMI; infertility type; period of infertility and number of previous IVF attempts were chosen in the propensity score matching (PSM) model to match 1374 women who taken the MPA+ hMG protocol with 134 women who received the MPA+ hMG+ LE treatment at a 1:1 ratio. FORT was selected as the primary outcome measure. The number of oocytes retrieved, viable embryos, hMG dosage, duration, oocyte maturity rate, fertilization rate, and implantation rate were established as secondary outcomes.

**Results:**

FORT was substantially elevated in the MPA+hMG+LE group compared with the MPA+hMG group (61% [35%, 86%] vs. 40% [25%, 60%], P <.001). Interestingly, the LE cotreatment group had a considerably lower mature oocyte rate despite having a similar number of mature oocytes and embryos recovered. The average hMG dosages and durations in the study group were similar to those in the control group. The implantation rate in the study group was numerically higher but without statistic significant than that in the control groups (43.15% (107/248) vs. 38.59% (115/298), OR 1.008, 95% CI 0.901-1.127; P >.05).

**Conclusion:**

The effect of LE combined with PPOS on FORT is better than the effect of the standard PPOS treatment in women with PCOS and a high BMI, but there is no substantially beneficial impact on pregnancy outcomes or the cycle features of COS, including consumption of hMG.

## Introduction

Polycystic ovary syndrome (PCOS) is a prevailing form of endocrinopathy that affects women of reproductive age (1). The rates of hyperandrogenism, obesity, and primary infertility have increased dramatically among women with PCOS over the last decade, resulting in a more severe phenotype among this population (2). Infertile women with PCOS may be treated with *in vitro* fertilization (IVF), laparoscopic ovarian surgery, and behavioural, and pharmaceutical interventions (including gonadotropins, metformin, aromatase inhibitors, and clomiphene citrate (CC)) (3). IVF is regarded as a third-line therapy and is often used in cases in which tubal factors and male factors exist (3).

As an alternative to standard GnRH analogues, progestin-primed ovarian stimulation (PPOS) by administering human menopausal gonadotropin (hMG) and medroxyprogesterone acetate (MPA) simultaneously from the early follicular phase successfully inhibits the oestradiol (E2)-induced LH surge (4). The PPOS protocol could achieve similar numbers of oocytes, viable embryos, and pregnancy outcomes (5); moreover, it is more patient-friendly, as it can further reduce the injection burden compare with the conventional GnRH analogue protocol (6).

Previous studies found that increased body mass index (BMI) is probably linked to an increased risk of insufficient follicle development as well as an increased follicle-stimulating hormone (FSH) requirement in the process of ovarian stimulation for IVF (7, 8) or dysregulation of meiotic spindle formation (9) and, consequently, developmental ability (10). Preliminary data showed that in normo-cycling women, the ratio of the preovulatory follicle count (PFC) to the antral follicle count (AFC), widely recognised as the follicular output rate (FORT), is positively linked to IVF outcomes (11, 12) and is regarded as a qualitative indicator of the follicular response. For women with PCOS, especially those who are obese, letrozole (LE) is recommended as a first-line treatment option for the induction of ovulation (13). LE can improve the follicular response to FSH by elevating intrafollicular androgen levels and reducing circulating oestrogen concentrations (14). At present, LE is extensively utilised as an adjunct for IVF treatment (15). Therefore, the current retrospective cohort study was conducted to evaluate the impact of combining LE with the PPOS protocol on FORT, as well as the features of the frozen embryo transfer (FET) cycle and oocyte pick-up cycle in high-BMI women with PCOS receiving IVF treatment.

## Materials and methods

### Patients and study setting

**Figure 1:**
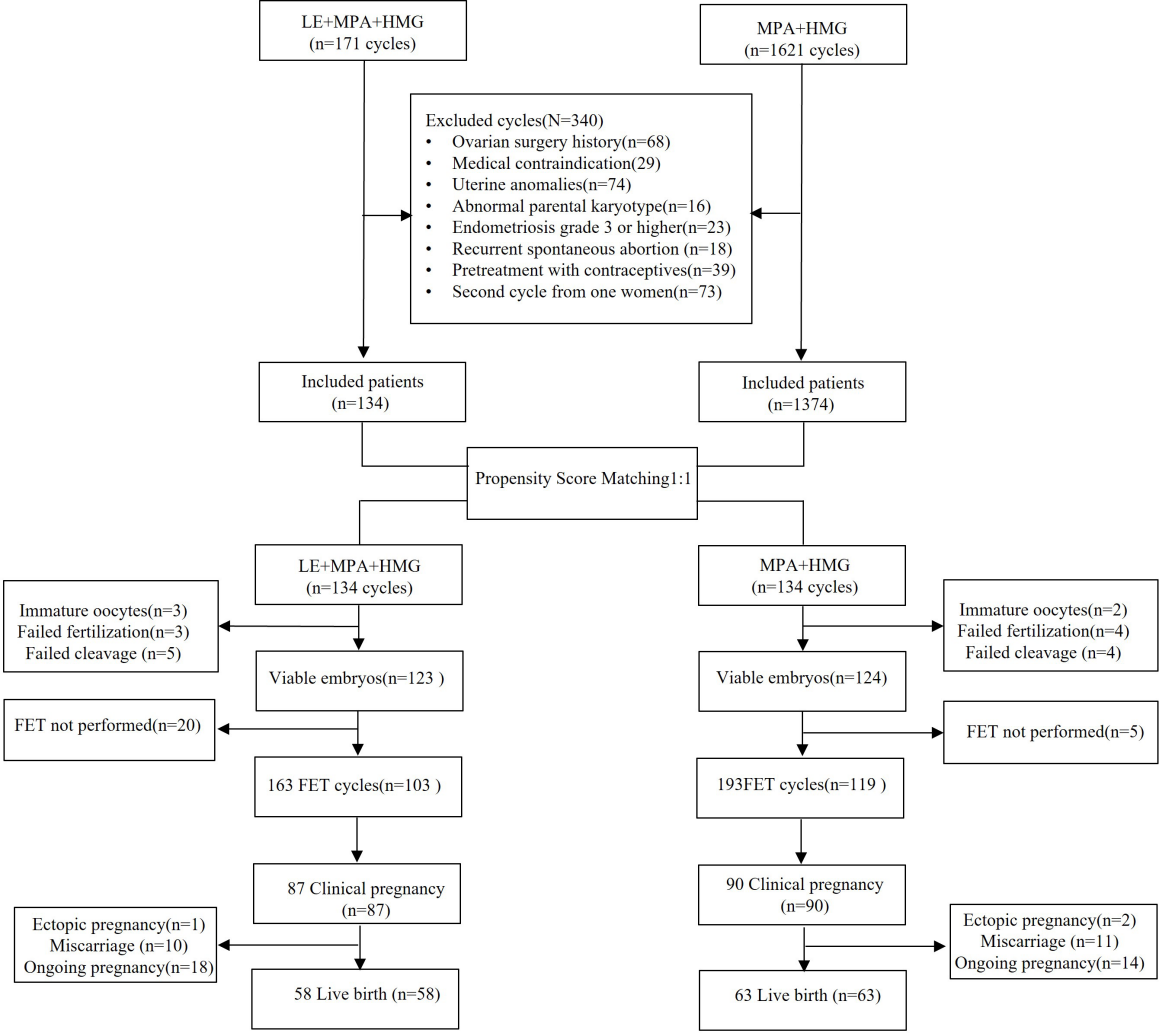
Flow chart of the study. IVF, *In vitro* fertilization; ICSI, Intracytoplasmic sperm injection; FET, Frozen embryo transfer; LE, Letrozole; MPA, Medroxyprogesterone acetate; hMG, Human menopausal gonadotropin.

The research protocol for this study was approved by the Shanghai Ninth People’s Hospital Ethics Committee (Institutional Review Board). Women with PCOS who completed IVF/ICSI cycles between January 2017 and September 2022 were recruited to the control group (hMG+MPA) and the study group (hMG+MPA+LE). For patients who received more than one cycle of COS within this time frame, only the first cycle was considered to prevent repeated inclusion. The patients satisfied the following conditions: 1. BMI between 25 and 37 kg/m2; 2. basal FSH level < 10 mIU/ml; 3. Age between 21 and 40 years; and 4. at most 1 previous cycle with no available embryo. According to the 2003 Rotterdam consensus (16), at least two of the following symptoms were required for women to be diagnosed with PCOS. 1) oligo- and/or anovulation; 2) ultrasonography appearance of polycystic ovaries; or 3) biochemical and/or clinical indicators of hyperandrogenism. Ultrasonography was unnecessary in cases where both hyperandrogenism and oligo- or anovulation existed. Reproductive, metabolic, and psychological factors were all considered in the assessment and management of the condition once diagnosed with PCOS. The exclusion criteria included the presence of the following disorders: hyperandrogenaemia and ovulatory dysfunction due to other aetiologies, such as thyroid disease, hyperprolactinaemia, androgen-secreting tumours, and congenital adrenal hyperplasia. Women who were currently receiving treatment for a clinical condition, for example diabetes or high blood pressure, were also eliminated. **Figure 1** depicts the study process.

### Controlled ovarian stimulation

Patients were given 150-225 IU/d hMG intramuscularly (Anhui Fengyuan Pharmaceutical Co., Ltd.) and 4 mg/d MPA orally (Shanghai Xinyi Pharmaceutical Co., Ltd.) from the third day of the menstrual cycle (MC3) until the trigger day. The study group was given oral LE (Jiangsu Hengrui Pharmaceutical Co., Ltd., 2.5 mg/day) starting on MC3 and continued this treatment for 5 days. Our research objects were women who have PCOS with high BMI, the median number of AFC was 20 and the mean BMI was 28 kg/m^2^. Except the number of AFC and BMI, the basal FSH value also been suggested as one of the influencing factors to select the initial Gn doses in IVF/ICSI treatment (17, 18). Women with basal FSH <7 mIU/ml were administered hMG 225 IU/day, for individuals with mildly increased basal FSH (7-10 mIU/ml) were administered hMG at a beginning dosage of 150 IU/day. From MC8 forwards, hMG dosages were modified for both groups every 2–4 days. If there were more than 20 follicles with a diameter >10 mm, we decreased the dosage of hMG from 225 to 150 IU. And if the FSH level was lower than 10 mIU/ml, we would add 75IU to the original dose. The hMG dose was adjust every 2–4 days according to the above principles.

When the dominant follicle reached a size exceeding 20 mm or more than three follicles reached a size exceeding 18 mm, 2000 IU or 5000 IU human chorionic gonadotropin (Lizhu Pharmaceutical Trading Co., China) in combination with 0.1 mg triptorelin (Decapeptyl; Ferring Pharmaceuticals, Germany) was employed to trigger the final phase of oocyte maturation. However, or 5000 IU human chorionic gonadotropin (hCG (500 IU hCG combined with 0.2 mg triptorelin or only 0.2 mg triptorelin was used if the patient was at risk of developing hyperstimulation. Under transvaginal ultrasound (TVS) guidance, oocytes were retrieved 34-38 hours after triggering. Aspiration was performed on all follicles measuring >10 mm in diameter (4).

Oocytes were then fertilised using conventional IVF or intracytoplasmic sperm injection (ICSI), depending on the quality of the semen and the success rate of previous fertilization attempts (19). The quantity and distribution of blastomeres, as well as the extent of fragmentation, were measured in the embryos. Within three days following oocyte retrieval, high-quality embryos (grade-1 and grade-2 6-cell embryos and above) were vitrified and frozen using the procedures stipulated by Cummins et al. (4, 20). Low-quality embryos were subjected to culture for a longer period, whereas blastocysts that were well-formed were frozen on day 5 or 6 (4).

### Measurement of *hormones*

On MC3, MC8, MC10-12, the day of the trigger and the day following the trigger, serum levels of LH, FSH, E2, and P4 were measured. Chemiluminescence (Abbott Biologicals B.V., the Netherlands) was used to analyse the hormone levels. The maximum E2 value that could be measured was 5000 pg/ml. Samples with an E2 concentration over 5000 pg/ml were recorded at a value of 5000 pg/ml. The sensitivity thresholds were as follows: P4 0.1 ng/ml; E2, 10 pg/ml; LH, 0.09 mIU/ml; and FSH, 0.06 mIU/ml.

### Preparation of endometrium and frozen embryo transfer

Following the procedures outlined previously (4, 21), the preparation of endometrium and FET were scheduled in the second cycle following oocyte retrieval. LE was initially prescribed for mild stimulation of the endometrium as our data showed superiority of this protocol over hormone replacement therapy (HRT) (22). Those who had trouble conceiving after undergoing mild stimulation cycles or who had a history of an abnormally thin endometrium (≤ 6 mm) were then subjected to HRT. The women who participated in the mild stimulation cycle received 2.5 or 5 mg of LE for 5 days, starting at MC3. Ovulation was induced by injecting urine hCG (5000 IU) under the following conditions: dominant follicle diameter ≥17 mm, endometrium lining ≥8 mm, P4 level ≤1 ng/ml, and E2 level ≥150 pg/ml. Two or three days later, progesterone was started, and then five days or seven days after ovulation induction, abdominal ultrasound was used to guide the transfer of day-3 embryos or day-7 blastocysts. We used the “freeze-all” strategy and there was a waiting period between oocyte retrieval and embryo transfer. Our recruitment period was from January 2017 to September 2022 and patients were followed up to January 9, 2023.

### Outcome measures

In this investigation, FORT was used as the primary outcome. The number of retrieved oocytes and viable embryos, the oocyte retrieval rate, the oocyte maturity and fertilization rates, the hMG dosage and duration, and the implantation rate were established as secondary outcomes. FORT was computed by using the ratio between the number of preovulatory follicles (PFCs) on hCG day × 100 and the number of AFCs at baseline. A prior study (12) concluded that only follicles between 16 and 22 mm in diameter should be included in the computation of FORT, to establish small antral follicles that responded best to FSH. The rate of oocyte retrieval was computed by dividing the total number of ruptured follicles by the sum of recovered oocytes. The rate of oocyte maturation was computed by dividing the sum of mature oocytes by the sum of all retrieved oocytes; To determine the fertilization rate, we divided the sum of fertilized oocytes by the sum of mature oocytes; the sum of fertilized oocytes was divided by cleaved embryos to obtain the cleavage rate. The rate of cycle cancellation was calculated as the sum of the number of patients whose oocyte retrieval resulted in zero viable embryos. At 4 weeks following FET, ultrasound detection of a gestational sac with or without foetal heart activity indicated the diagnosis of clinical pregnancy. The clinical pregnancy rate was determined by dividing the total number of clinical pregnancies by the sum of FET cycles. The implantation rate was computed by dividing the sum of embryo transfers by the total number of gestational sacs. The miscarriage rate was determined by the percentage of pregnancies that ended early due to therapeutic or spontaneous abortions.

### Statistical *analysis*

To account for inherent disparities in the baseline characteristics of the two groups, we developed a propensity score matching (PSM) model. Ten variables were chosen for use in the propensity score estimation, including, AFC; age; basal levels of P4, E2, LH, and FSH; BMI; infertility type (primary or secondary); period of infertility and number of previous IVF attempts (0, 1–2 or ≥3). We used the nearest neighbour random matching technique to match patients receiving MPA+hMG+LE protocol with those receiving MPA+hMG treatment in a 1: 1 ratio. PSM was completed utilising R software (version 4.0.3; R Foundation for Statistical Computing, Vienna, Austria).

The mean ± standard deviation (SD) and Student’s t test were used to display and evaluate normally distributed continuous data. The median [25th percentile, 75th percentile] and Mann–Whitney U tests were used to express and assess nonnormally distributed continuous data. Categorical data are displayed as n (percentage) and compared with Fisher’s exact test or Pearson’s chi-squared test. The SPSS statistical package (version 24, SPSS Inc.) was utilised for data analysis. Two-sided P <.05 was the criterion for statistical significance.

As a patient may have more than one FET cycle, we used the generalised estimating equation (GEE) modelling to reduce the potential bias of repeated cycles and compared the different treatment protocols with the odds ratio (OR) and corresponding 95% confidence interval (CI). P <.05 was the criterion for statistical significance. Variables as the clinical pregnancy rate, implantation rate, miscarriage rate, ectopic pregnancy rate and live birth rate were included in the regression equation.

## Results

### Patient *features*

The study’s flow chart is summarised in **Figure 1**. Specifically, we enrolled 1792 high-BMI (BMI>25) women with PCOS who were candidates for assisted reproductive technology treatment in our clinical centre between January 2017 and September 2022. Based on the criteria outlined in the Methods and Materials, 340 cycles were eliminated. For the remaining 1508 women, we used the nearest neighbour random matching method to match 1374 women who received the MPA+hMG+LE protocol with 134 women who received the MPA+hMG treatment at a 1:1 ratio. The baseline and outcome parameters before PSM are presented in **Table 1**. Although our patients were recruited from January 2017 to September 2022, but the number of patients enrolled in 2022 only accounted for less than 7% of the total number due to the COVID-19 epidemic in China. Women who received the MPA+hMG+LE protocol were concentrated in the year of 2020 and 2021, while those received the MPA+hMG treatment were evenly distributed during the enrolment period. Our follow-up time was up to January 9, 2023, hence 93% of the patients had at least one year of follow-up after recruitment. Recruitment trend of patients in different years before and after PSM are provided in **Supplemental Figure 1**. All patients postmatching finished their oocyte retrieval cycles and were successful in obtaining oocytes (ranging from 1 to 52), however, 21 of these patients did not have any viable embryos. In addition, 222 of the remaining 247 patients finished 356 FET cycles. Postmatching analysis showed no significant variations in any baseline characteristics across the groups (all P >.05) (**Table 1**).

**Table 1:** The baseline parameters of the two groups before and after PSM.

Parameters	Pre-match		P value	Post-match		P value
	Study group:	Control group:		Study group:	Control group:	
	hMG+MPA+LE (n=134)	hMG+MPA (n=1374)		hMG+MPA+LE (n=134)	hMG+MPA (n=134)	
**Age (years)**	32.43 ± 3.58	35.36 ± 4.08	<0.001	32.43 ± 3.58	32.35 ± 3.7	0.854
**Duration of infertility (years)**	4.04 ± 2.33	4.2 ± 2.42	0.52	3.7 ± 2.7	3.93 ± 2.91	0.501
**Primary infertility, n (%)**	64.93% (87/134)	22.56% (310/1374)	<0.001	64.93% (87/134)	62.69% (84/134)	0.703
Indication, n (%)			<0.001			0.884
**Male factor**	14.93% (20/134)	19.65% (270/1374)		14.93% (20/134)	13.43% (18/134)	
**Tubal factor**	65.67% (88/134)	50.15% (689/1374)		65.67% (88/134)	67.91% (91/134)	
**Combination of factors**	8.21% (11/134)	21.11% (290/1374)		8.21% (11/134)	9.70% (13/134)	
**Unknown factor**	11.19% (15/134)	9.1% (125/1374)		11.19% (15/134)	8.96% (12/134)	
Previous IVF failure			<0.001			0.905
**0**	88.80% (119/134)	65.28% (897/1374)		88.80% (119/134)	90.30% (121/134)	
**1–2**	8.96% 12/134)	21.83% (300/1374)		8.96% (12/134)	7.46% (10/134)	
**> 3**	2.24% (3/134)	12.88% (177/1374)		2.24% (3/134)	2.24% (3/134)	
**BMI**	28.44 ± 2.65	30.12 ± 90.97	<0.001	28.44 ± 2.65	28.18 ± 2.58	0.411
Basal hormone concentrations						
**FSH (IU/L)**	5.23 ± 1.28	5.23 ± 1.38	0.768	5.23 ± 1.28	5.26 ± 1.5	0.848
**LH (IU/L)**	4.12 ± 2.18	4.01 ± 2.43	0.132	4.24 ± 2.6	4.07 ± 2.29	0.583
**E2 (pg/ml)**	33.34 ± 11.79	32.26 ± 12.56	0.253	33.34 ± 11.79	33.25 ± 12.35	0.991
**P (ng/ml)**	0.23 ± 0.1	0.23 ± 0.11	0.682	0.23 ± 0.13	0.22 ± 0.13	0.217
**AFC**	19.64 ± 6.99	17.82 ± 6.49	0.002	20 [15,22]	20 [16,20.25]	0.774

Data are presented as mean ± standard deviation and median [25th percentile, 75th percentile]. BMI, Body Mass Index; FSH, Follicle stimulating Hormone; LH, Luteinizing Hormone; E2, Estrogen; P, Progesterone; AFC, Antral Follicle Counting.

### Ovarian stimulation, follicle development, and oocyte performance

The study and control groups exhibited similar numbers of retrieved oocytes (13.5 [8.75, 20.25] vs. 15 [9, 21], P >.05) and viable embryos (5 [2, 8] vs. 5 [2.75, 8], P >.05). Neither the hMG doses nor the period of ovarian stimulation were significantly different between the two groups (P >.05). The sums of follicles with diameters of 10-12 mm and 12-14 mm were comparable across the two groups. The study group had a substantial reduction in the sum of follicles with a diameter of 14-16 mm (2 [0, 6] vs. 3 [1, 6], P <.05) but significantly higher number of follicles larger than 16 mm when compared with the control group (10.5 [7, 18] vs. 8 [4, 11], P <.05) (**Table 2**). Consistent with the above results, FORT was substantially elevated in the study group compared with the control group (61% [35%, 86%] vs. 40% [25%, 60%], P <.01). Although the mature oocyte rate was meaningfully reduced in the LE cotreatment group (81% ± 18% vs. 86% ± 12%, P <.05), the oocyte retrieval rate was similar in the two groups. Additionally, the number of aspirated follicles and mature oocytes were not significantly different. Of the 134 women receiving LE+MPA+HMG treatment, 3 had no mature oocytes, 3 failed fertilization, and 5 failed cleavage. Of the 134 women who underwent the MPA+HMG protocol, 2 had no mature oocytes, 4 failed fertilization, and 4 failed cleavage. The cycle cancellation rates for unviable embryos that were not significantly different between the two groups (8.2% (11/134) vs. 7.5% (10/134), P >.05) (**Table 2**).

**Table 2:** COS characteristics and outcomes after PSM.

	Study group: hMG+MPA+LE (n=134)	Control group: hMG+MPA (n=134)	P value
**hMG dose (IU)**	2370.15 ± 738.98	2392.16 ± 789.52	0.841
**hMG duration (d)**	9.31 ± 1.86	9.63 ± 2.4	0.391
**hCG Dose on trigger day (IU)**	2082.09 ± 1255.36	2221.64 ± 1637.68	0.989
**GnRHa Dose on trigger day (mg)**	0.14 ± .05	0.12 ± 0.04	0.000
**10-12-mm follicles on hCG day (n)**	2 [0,4]	2 [0,5]	0.143
**12-14-mm follicles on hCG day (n)**	2 [0,5.25]	3 [1,6]	0.278
**14-16-mm follicles on hCG day (n)**	2 [0,6]	3 [1,6]	0.033
**> 16-mm follicles on hCG day (n)**	10.5 [7,18]	8 [4,11]	0.000
**FORT (%)**	61 [35,86]	40 [25,60]	0.000
**Punctured follicles (n)**	18 [12,29]	20 [13,27.25]	0.555
**Oocyte retrieved (n)**	13.5 [8.75,20.25]	15 [9,21]	0.777
**Mature oocytes (n)**	11 [7,16]	12.5 [7,17]	0.149
**Fertilized oocytes (n)**	9 [5,13]	10 [6,14]	0.290
**Cleaved embryos (n)**	9 [5,12.25]	9.5 [5.75,13]	0.310
**High-quality embryos (n)**	3 [2,6]	4 [2,7]	0.370
**Blastocyst embryos (n)**	1 [0,3]	1 [0,2]	0.178
**All cryopreserved embryos (n)**	5 [2,8]	5 [2.75,8]	0.756
**Oocyte retrieval rate (%)**	75% ± 19%	74% ± 22%	0.904
**Mature oocyte rate (%)**	81% ± 18%	86% ± 12%	0.040
**Fertilization rate (%)**	79% ± 18%	79% ± 16%	0.749
**Cleavage rate (%)**	96% ± 11%	97% ± 7%	0.907
**Cycle cancellation rate (%)**	8.2% (11/134)	7.5% (10/134)	0.820

Data are presented as mean ± standard deviation and median [25th percentile, 75th percentile] or number (percentage). All the value of (n) were calculated per cycle.

### Profiles of hormones during treatment

**Figure 2:**
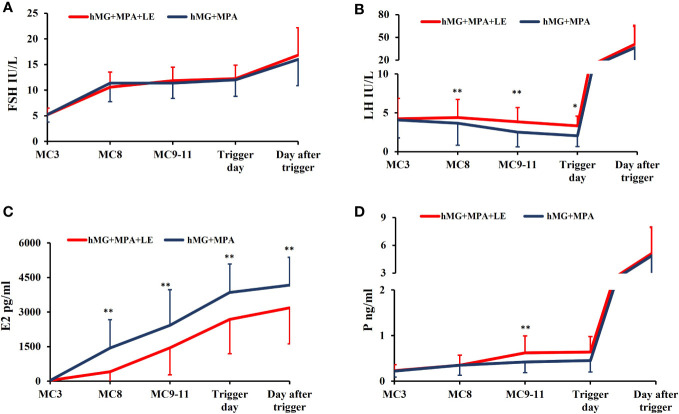
The dynamic changes in hormones during ovarian stimulation in the two groups. **(A)**. Serum FSH levels in the two groups during COS. **(B)**. Serum LH concentration in the two groups during COS. **(C)**. Serum E2 level in the two groups during COS. **(D)** Serum P levels in the two groups during the COS. The solid red lines represent the study group (hMG+MPA+LE) and the solid heavy blue lines represent the control group (hMG+MPA). The asterisks denote significant changes in hormone levels at the indicated time points (*P <.05, **P <.01).

**Figure 2** depicts the endocrine dynamics that occurred in response to ovarian stimulation, including those of P4, E2, LH, and FSH. After the hMG injection, FSH levels spiked considerably 5 days later and then remained constant until the trigger day. Rapid elevation of FSH to over 15 mIU/ml was observed following the dual trigger. No remarkable differences were observed in FSH levels between the two groups at any time point (**Figure 2A**).

LH remained low in both groups throughout the COS. There was a declining trend in LH levels in the control group. On the other hand, the LH concentration in the study group was rather stable for the initial five days. During COS, LH levels at MC8, MC9-11 and the trigger day were remarkably higher in the study group than in the control group(P <.01). Neither group had any cases of premature LH surge (**Figure 2B**).

As several follicles matured, there was a continuous rise in serum E2. Due to the use of LE, the oestrogen values in the study group were lower than those in the control group during COS, and the variation was statistically significant at all time points (P <.01) (**Figure 2C**).

P4 levels in both groups gradually increased during ovulation stimulation. Additionally, the P4 levels in the study group were substantially elevated compared with those in the control group at MC9-11(P <.01) (**Figure 2D**).

### Outcomes of pregnancies following FET procedures

There were 247 women with viable embryos that developed successfully. A total of 546 embryos were thawed, and all (100%) were viable after the thawing procedure. Ultimately, 356 FET cycles were completed by 222 women. In the LE cotreatment group, 103 women finished 163 FET cycles in total: 63 women had accomplished one FET cycle, 26 women had completed two FET cycles, 14 women had accomplished more than or equal to three FET cycles. Whereas 119 women in the PPOS group finished 193 FET cycles: including 65 women with one FET cycle, 38 women with two FET cycles, 16 women finished greater than or equal to three FET cycles. In total, 86% of patients in both groups had fewer than three FET cycles. However, 46 women failed to start their FET cycles for numerous reasons in the two groups. There was no significant difference on the stage and number of embryos transferred per cycle between the two groups, and the neonatal status between the two groups were similar (P >.05) (**Table 3**).

**Table 3:** Pregnancy outcomes after FET.

Variable	Study group:	Control group:			P value
	hMG+MPA+LE	hMG+MPA			
**Patients (n)**	103	119			
**FET cycles (n)**	163	193			
**Thawed embryos (n)**	248	298			
**Viable embryos after thawed (n)**	248	298			
**The number of embryos per transfer (n)**	1.53 ± 0.50	1.54 ± 0.50			0.758
**Indication, n (%)**					0.497
**cleavage-stage embryo**	56.85% (141/248)	59.73% (178/298)			
**blastocyst embryo**	43.15% (107/248)	40.27% (120/298)			
**Endometrial preparation n (%)**					0.605
**Mild stimulation**	63.80% (104/163)	61.14% (118/193)			
**Hormone therapy**	36.20% (59/163)	38.86% (75/193)			
**Endometrial thickness (mm)**	10.44 ± 2.56	10.09 ± 2.04			0.249
Newborn					
**Single birth (n)**	47	56			
**Single birthweight (g)**	3399.57 ± 565.19	3304.17 ± 531.81			0.403
**Twin birth (n)**	11	9			
**Twin birthweight (g)**	2535 ± 392.22	2310 ± 495.71			0.117
**Variable adjusted in GEE models**			OR	95% CI	
**Clinical pregnancy rate per transfer (%)**	53.37% (87/163)	52.85% (102/193)	1.008	0.901-1.127	0.891
**Implantation rate (%)**	43.15% (107/248)	38.59% (115/298)	1.065	0.918-1.235	0.405
**Miscarriage rate (%)**	13.79% (12/87)	15.69% (16/102)	0.982	0.891-1.083	0.714
**Ectopic pregnancy rate (%)**	1.15% (1/87)	1.96% (2/102)	0.987	0.95-1.026	0.514
**Live birth rate per cycle (%)**	35.58% (58/163)	34.20% (66/193)	0.991	0.838-1.171	0.913
**Live birth rate per patient (%)**	56.31% (58/103)	60.55% (66/119)	0.991	0.838-1.171	0.913

Data display as mean ± SD or number (percentage). Pregnant data were followed up until January 9, 2023.

Our results showed that after controlling for the potential bias of repeated cycles from one patient, the different treatment protocols in the LE cotreatment group and the PPOS group were not associated with significant differences in the indicators as implantation rates (43.15% (107/248) vs. 38.59% (115/298), OR 1.008, 95% CI 0.901-1.127; P >.05), clinical pregnancy rate, miscarriage rate, and live births rate (P >.05). (**Table 3**). All pregnancy data were followed up until January 9, 2023 (**Table 3**).

## Discussion

This research showed that LE may play a role as an adjuvant medicine to enhance the FORT of the PPOS regimen in PCOS patients with a high BMI. Nevertheless, the number of retrieved oocytes and mature oocytes, the dose of gonadotropin consumption and gonadotropin days, and the number of embryos in the combined group were similar to those in the group that received the PPOS protocol alone. The implantation rate was elevated in the combined group compared with the PPOS group, but the difference was not significant, and the rates of clinical pregnancies, miscarriages and live births were comparable between the two groups.

Increased BMI in PCOS is linked to elevated androgen levels, which could block dominant follicle development and cause follicular degeneration (23). In our study, the FORT in the LE cotreatment group was significantly greater than that in the control group. The higher FORT after LE combination therapy may be the result of endocrine alterations. Coadministration of LE causes an acute hypoestrogenic condition, which relieves the hypothalamic-pituitary axis of oestrogenic negative responses and enhance gonadotropins production (24), these changes may explain the increased follicle diameter at oocyte retrieval time in the LE cotreatment group. We found that the number of larger follicles (>16 mm) was substantially elevated in the study group compared with the control group. After diameter deviation, increased LH levels tend to promote dominant follicle selection and enhance the development of dominant follicles (25, 26). Notably, the dose of GnRH-a for triggering was markedly enhanced and the dose of hCG was decreased without statistical difference in the LE cotreatment group compared with the PPOS-only group. An increased number of large preovulatory follicles was observed in the LE cotreatment group, suggesting that women in that group had a higher likelihood of receiving a single GnRHa trigger rather than a dual trigger. The number of oocytes retrieved and the oocyte retrieval rate were similar between the two groups, indicating that the ovulation trigger method in the present study did not affect oocyte retrieval.

Although LH values were meaningfully increased in letrozole cotreatment group compared with the standard PPOS group, FSH levels were similar between the two group. Women with PCOS would have a partial pituitary desensitization and relative decline of FSH responsiveness (27, 28) which might owe to the hyperactive GnRH neurons (29). Higher LH values in the study group might induced by LE through blocking oestrogen production (24). Progestogen was one of the precursors of estrogens and transformed into estrogens by aromatase (30). LE, an aromatase inhibitor, can inhibit the production of estrogens, thereby reducing estrogens level and accumulating progesterone (15, 24, 30). Hence, lower serum E2 levels and higher P levels were observed after cotreatment with LE in the PPOS protocol.

Notably, oocyte maturity rates were significantly decreased in the LE cotreatment group compared with the PPOS-only group, although mature oocyte yields were comparable. The influence of LE on oocyte maturity remains controversial in the literature (31–33). LE, an aromatase inhibitor, is usually used in patients who need fertility preservation, such as those with breast cancer, to reduce oestrogen levels (33). Our research is consistent with Quinn’s study showing that GnRH antagonist protocol cotreatment with LE in breast cancer patients decreased the oocyte maturity rate (33). LE coadministration decreased oestrogen levels and resulted in the accumulation of progesterone, 17α-progesterone and testosterone (15, 24, 30), These changes in the endocrine microenvironment affect meiotic maturation probably by inhibiting oocyte cytoplasmic maturation, contributing to reduced oocyte maturation. There is also evidence that LE does not increase the risk of spindle assembly and preimplantation developmental arrest (34). Hu’s study of mouse oocytes showed that the antral space formed earlier if they were cultured in the presence of aromatase inhibitor, while the oocyte competency was not reduced (35). In the current retrospective study, lead follicles were trigger at diameters of 18 mm, leading to lower oocyte maturity rates in the LE cotreatment group. Hence, Oktay (32) suggested that instead of triggering lead follicles at diameters of 17–18 mm, they should trigger at 19.5–20.5 mm under LE-containing stimulation, as LE cotreatment requires a different trigger criterion. In the present research, the lower oocyte maturity rates in the LE cotreatment group did not affect the number of mature oocytes as the number of large follicles on the trigger day was significantly higher. Prospective randomised controlled studies with different trigger criteria for the PPOS- LE cotreatment protocol are needed in the future.

In our present study, FORT didn’t convert into significant higher implantation rate here. Although previous studies illustrated that patients with an elevated FORT can achieve improved clinical outcomes (36) and good pregnancy outcomes (37) in IVF cycles and that embryos generated from oocytes from the dominant follicle group could have enhanced implantation potential since they are less fragmented (38, 39), opposing studies have linked an abundance of dominant follicles to impaired oocyte growth performance and a poor pregnancy rate, since excessive follicular development during ovarian stimulation might cause oocyte overmaturation, leading to unsuccessful pregnancies (40). We have showed that oocyte maturity rates were significantly decreased in the LE cotreatment group compared with the PPOS-only group in the previous statement, and LE-containing stimulation should trigger lead follicles at larger diameters than the original standard (32). Therefore, we speculated that although the proportion of FORT increased significantly after adding LE in PPOS protocol, its oocyte development potential did not increase significantly. However, this needs to be confirmed by large-scale prospective randomized controlled studies.

A higher BMI has been linked to a greater need for gonadotrophins during stimulation (7). PCOS may be accompanied by an altered ovarian response to gonadotropins, which would reduce early ovarian responsiveness compared with that in ovulatory controls (41). FORT is an objective method for determining the real effect of external FSH on follicles since it is not impaired by preexisting antral follicle population size (11). Although concurrent LE administration enhanced FORT in the PPOS protocol, no significant difference in gonadotropin consumption was observed. This was surprising given that previous studies have suggested that LE lowers the overall gonadotropin intake for ovarian stimulation (24, 42, 43). In contrast to low responders, high responders with PCOS exhibit FSH receptor upregulation in their follicular granulosa cells (44), which could explain why LE did not work to lower the overall gonadotropin dosage necessary for ovarian stimulation in hyperresponders (45).

Our study’s retrospective nature and limited sample size are notable drawbacks. As a retrospective study, there existed selectivity bias in this research. Most Chinese patients with PCOS (80%) are within the healthy weight range (2). Additionally, our data were acquired from a single centre; as a result, it was challenging to gather sufficient patient data to detect statistically significant variations. There is also the possibility of unmeasured or unidentified confounders in this retrospective analysis, which might result in less-than-perfect matching and weaken the reliability of the results. In addition, despite having embryos of high quality, some patients were unable to finish their FET cycles for a variety of reasons. Thus, the present research should be considered a preliminary effort, and the development of additional evidence requires further by more prospective studies to validate the impact of combine LE with PPOS for women with PCOS women and a high BMI. Further research on the application of LE in women with PCOS and different BMIs is needed to help physicians to develop individualised treatment plans for each patient.

## Conclusion

Our findings show that the addition of LE to the PPOS regimen increased FORT. In patients with PCOS and high BMI who were receiving IVF therapy, there was not a statistically favourable impact of LE addition on the cycle parameters of COS, including hMG consumption, or on pregnancy outcomes.

## Data availability statement

The original contributions presented in the study are included in the article/**Supplementary Material**. Further inquiries can be directed to the corresponding authors.

## Ethics statement

The studies involving human participants were reviewed and approved by The Ethics Committee of Shanghai Ninth People’s Hospital (Institutional Review Board) (Number: SH9H-2021-T294–1). Written informed consent for participation was not required for this study in accordance with the national legislation and the institutional requirements.

## Author contributions

YK and QZ conceived and direct this study. XS and JL contribute to data collection and analysis. YL dedicated to draft and revise the manuscript. All authors have made a corresponding contribution to this article. The authors have nothing to declare on the final manuscript. All authors contributed to the article and approved the submitted version.
