# Supplementary figures and images for: Letrozole cotreatment improves the follicular output rate in high-body-mass-index women with polycystic ovary syndrome undergoing IVF treatment
Source: Front Endocrinol (Lausanne). 2023 Mar 3;14:1072170. doi: 10.3389/fendo.2023.1072170 (PMC10020617; doi:10.3389/fendo.2023.1072170)

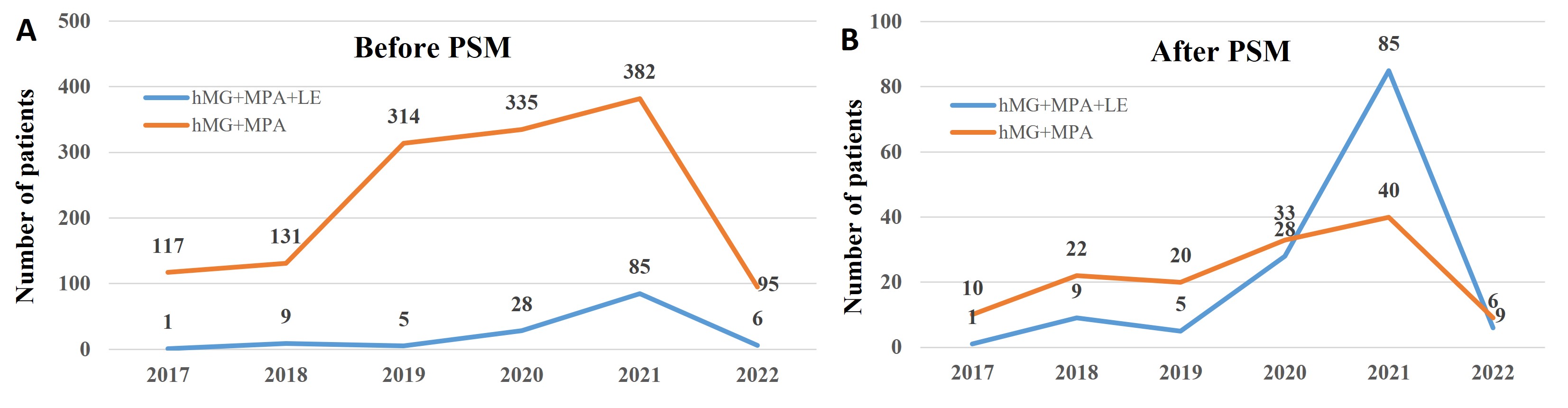

Supplement: Supplementary file 1 [file Image_1.jpeg]
