# Supplementary material for: Letrozole cotreatment improves the follicular output rate in high-body-mass-index women with polycystic ovary syndrome undergoing IVF treatment
Source: Front Endocrinol (Lausanne). 2023 Mar 3;14:1072170. doi: 10.3389/fendo.2023.1072170 (PMC10020617; doi:10.3389/fendo.2023.1072170)
Supplement: Supplementary file 2 [file Table_1.docx]

**Supplement table 1. The baseline and outcome parameters of the two groups before PSM.**

|  | **Study group:** | **Control group:** | P value |
| --- | --- | --- | --- |
|  | **hMG+MPA+LE** | **hMG+MPA** |  |
| **No.** | 134 | 1374 |  |
| **Age (years)** | 32.43±3.58 | 35.36±4.08 | 0.000 |
| **Duration of infertility (years),** | 4.04±2.33 | 4.2±2.42 | 0.520 |
| **Primary infertility, n (%)** | 64.93% (87/134) | 22.56% (310/1374) | 0.000 |
| **Indication, n (%)** |  |  | 0.001 |
| **Male factor** | 20 | 177 |  |
| **Tubal factor** | 88 | 791 |  |
| **Combination of factors** | 11 | 313 |  |
| **Unknown factor** | 15 | 93 |  |
| **BMI** | 28.44±2.65 | 30.12±90.97 | 0.000 |
| **Basal hormone concentrations** |  |  |  |
| **FSH (IU/L)** | 5.23±1.28 | 5.23±1.38 | 0.768 |
| **LH (IU/L)** | 4.12±2.18 | 4.01±2.43 | 0.132 |
| **E2 (pg/ml)** | 33.34±11.79 | 32.26±12.56 | 0.253 |
| **P (ng/ml)** | 0.23±0.1 | 0.23±0.11 | 0.682 |
| **AFC** | 19.64±6.99 | 17.82±6.49 | 0.002 |
| **hMG duration (d)** | 9.59±1.86 | 9.7±2.06 | 0.059 |
| **hMG dose (IU)** | 2370.15±738.98 | 2257.9±645.27 | 0.070 |
| **hCG Dose on trigger day (IU)** | 1705.15±1146.85 | 1778.20±1106.82 | 0.024 |
| **GnRHa Dose on trigger day (mg)** | 0.12±0.04 | 0.11±0.04 | 0.001 |
| **>14-mm follicles on hCG day (n)** | 14.5 [9, 21] | 11 [7, 16] | 0.001 |
| **Punctured follicles (n)** | 18 [12, 29] | 18 [11, 26] | 0.140 |
| **Oocyte retrieved (n)** | 13.5 [8.75, 20.25] | 12 [6, 18] | 0.001 |
| **Mature oocytes (n)** | 11 [7, 16] | 9 [5, 15] | 0.043 |
| **Fertilized oocytes (n)** | 9 [5, 13] | 8 [4, 12] | 0.057 |
| **Cleaved embryos (n)** | 9 [5, 12.25] | 7 [4, 12] | 0.065 |
| **High-quality embryos (n)** | 3 [2, 6] | 3 [1, 6] | 0.202 |
| **Blastocyst embryos (n)** | 1 [0, 3] | 0 [0, 2] | 0.000 |
| **All cryopreserved embryos (n)** | 5 [2, 8] | 4 [2, 7] | 0.039 |
| **Oocyte retrieval rate (%)** | 0.75±0.19 | 0.68±0.23 | 0.001 |
| **Mature oocyte rate (%)** | 0.81±0.18 | 0.85±0.17 | 0.000 |
| **Fertilization rate (%)** | 0.81±0.16 | 0.82±0.18 | 0.092 |
| **Cleavage rate (%)** | 0.97±0.11 | 0.98±0.09 | 0.056 |
| **Cycle cancellation rate (%)** | 8.21% (11/134) | 8.94% (127/1421) | 0.777 |
| **FET Patients (n)** | 103 | 1153 |  |
| **FET cycles (n)** | 163 | 1752 |  |
| **Thawed embryos (n)** | 248 | 3016 |  |
| **Viable embryos after thawed (n)** | 248 | 3016 |  |
| **Clinical pregnancy rate per transfer (%)** | 53.37% (87/163) | 48.05% (842/1752) | 0.194 |
| **Implantation rate (%)** | 43.15% (107/248) | 35.67% (1076/3016) | 0.019 |

Note: Data are presented as median [25th percentile, 75th percentile].
