# Supplementary material for: Letrozole cotreatment improves the follicular output rate in high-body-mass-index women with polycystic ovary syndrome undergoing IVF treatment
Source: Front Endocrinol (Lausanne). 2023 Mar 3;14:1072170. doi: 10.3389/fendo.2023.1072170 (PMC10020617; doi:10.3389/fendo.2023.1072170)
Supplement: Supplementary file 3 [file Table_2.docx]

**Supplementary Table 2. Pregnancy outcomes of patient underwent the first cycle of IVF and transplantation from the two groups.**

| **Outcome** | **Study group** | **Control group** | **P value** |
| --- | --- | --- | --- |
|  | **(hMG+MPA+LE)** | **(hMG+MPA)** |  |
| Patients (n) | 48 | 60 |  |
| FET cycles (n) | 48 | 60 |  |
| Thawed embryos (n) | 48 | 60 |  |
| Viable embryos after thawed (n) | 48 | 60 |  |
| Clinical pregnancy rate |  |  |  |
| Per cycle (%) | 52.08 (25/48) | 48.33 (29/60) | 0.847 |
| Implantation rate (%) | 47.92 (23/48） | 38.33(23/60) | 0.335 |
| livebirth rate (%) | 39.58 (19/48） | 33.33(20/60) | 0.549 |

Note: Data are presented as mean ± standard deviation or number (percentage). Pregnant data were followed up until 1 December 2022.
