# Supplementary material for: Letrozole cotreatment improves the follicular output rate in high-body-mass-index women with polycystic ovary syndrome undergoing IVF treatment
Source: Front Endocrinol (Lausanne). 2023 Mar 3;14:1072170. doi: 10.3389/fendo.2023.1072170 (PMC10020617; doi:10.3389/fendo.2023.1072170)
Supplement: Supplementary file 4 [file Table_3.docx]

**Supplementary Table3. Baseline and COS characteristics of PCOS women with different BMI undergoing IVF/ICSI and outcomes**

|  | **High BMI:** | **Normal BMI:** | **Low BMI:** | **P** |
| --- | --- | --- | --- | --- |
|  | **hMG+MPA+LE (n=154)** | **hMG+MPA+LE (n=199)** | **hMG+MPA+LE (n=23)** | **value** |
| **Age (years)** | 31.81±3.61 | 31.34±3.78 | 31.52±2.33 | 0.422 |
| **Duration of infertility (years),** | 4.16±2.55 ^ab^ | 3.29±2.07 ^b^ | 4.8±1.99 ^a^ | 0.000 |
| **Primary infertility, n (%)** | 66.83% (103/154) | 66.33% (132/199) | 60.87% (14/23) | 0.850 |
| **Previous IVF failure** | 0.21±0.77 | 0.35±1.03 | 0.26±0.69 | 0.348 |
| **BMI** | 28.71±2.96 ^a^ | 21.91±1.93 ^b^ | 17.42±0.72 ^c^ | 0.000 |
| **Basal hormone concentrations** | |  |  |  |
| **FSH (IU/L)** | 5.19±1.28 ^b^ | 5.24±1.36 ^b^ | 6.35±1.73 ^a^ | 0.003 |
| **LH (IU/L)** | 4.57±2.45 | 5.55±3.66 | 5.99±3.55 | 0.074 |
| **E2 (pg/ml)** | 33.57±11.62 | 37.41±13.92 | 38.04±15.37 | 0.063 |
| **P (ng/ml)** | 0.21±0.11 ^b^ | 0.26±0.13 ^a^ | 0.24±0.12 ^ab^ | 0.004 |
| **AFC** | 21.7±7.46 | 20.26±5.28 | 18.7±3.88 | 0.094 |
| **hMG duration (d)** | 9.62±2.03 ^a^ | 8.64±1.36 ^b^ | 8.26±0.81 ^b^ | 0.000 |
| **hMG dose (IU)** | 2411.04±757.31 ^a^ | 1761.18±516.37 ^b^ | 1291.3±203.47 ^b^ | 0.000 |
| **10-12-mm follicles on hCG day (n)** | 22 [15.75, 30] | 24 [17, 31] | 20 [15, 31] | 0.291 |
| **12-14-mm follicles on hCG day (n)** | 19 [13, 28] | 24 [15, 31] | 23 [14, 31] | 0.198 |
| **14-16-mm follicles on hCG day (n)** | 16 [11, 23] | 18 [13, 25] | 13 [6, 29] | 0.073 |
| **> 16-mm follicles on hCG day (n)** | 10 [7, 20] | 15 [9, 20] | 14 [8, 31] | 0.185 |
| **Punctured follicles (n)** | 21 [13.75, 30] ^b^ | 24 [18, 33] ^a^ | 21 [12, 33] ^b^ | 0.019 |
| **Oocyte retrieved (n)** | 15 [10, 20.25] ^b^ | 18 [12, 24] ^a^ | 16 [10, 25] ^ab^ | 0.004 |
| **Mature oocytes (n)** | 11 [7, 17] ^b^ | 14 [9, 19] ^a^ | 12 [8, 20] ^ab^ | 0.028 |
| **Fertilized oocytes (n)** | 9 [5, 14] ^b^ | 12 [7, 15] ^a^ | 9 [5, 16] ^ab^ | 0.012 |
| **Cleaved embryos (n)** | 9 [5, 13.25] ^b^ | 11 [7, 15] ^a^ | 9 [5, 16] ^ab^ | 0.009 |
| **High-quality embryos (n)** | 4 [2, 7] | 5 [3, 8] | 4 [1, 6] | 0.313 |
| **Blastocyst embryos (n)** | 1 [0, 3] | 2 [0, 4] | 1 [0, 2] | 0.100 |
| **All cryopreserved embryos (n)** | 5 [2, 8] | 6 [3, 9] | 5 [2, 8] | 0.097 |
| **Oocyte retrieval rate (%)** | 0.71±0.22 | 0.73±0.21 | 0.82±0.17 | 0.092 |
| **Mature oocyte rate (%)** | 0.81±0.2 | 0.78±0.19 | 0.79±0.2 | 0.146 |
| **Fertilization rate (%)** | 0.8±0.19 | 0.83±0.14 | 0.79±0.16 | 0.462 |
| **Cleavage rate (%)** | 0.97±0.1 | 0.98±0.06 | 0.97±0.08 | 0.160 |
| **Cycle cancellation rate (%)** | 7.14% (11/154) | 5.52% (11/199) | 13.04% (3/23) | 0.372 |

Note: Data are presented as mean ± standard deviation and median [25th percentile, 75th percentile] or number (percentage). All the value of (n) were calculated per cycle.
